# Reappraisal of the Genetic Diversity Patterns in *Puya raimondii*—The Queen of the Andes: Insights from Molecular Marker Analysis Reveal an Inbreeding Reproductive Strategy

**DOI:** 10.3390/plants14030321

**Published:** 2025-01-22

**Authors:** Samela Draga, Sergio Sgorbati, Gianni Barcaccia

**Affiliations:** 1 Laboratory of Genetics and Genomics for Plant Breeding, Department of Agronomy Food Natural Resources Animals and Environment (DAFNAE), School of Agricultural Science and Veterinary Medicine, University of Padova, 35020 Padova, Italy; samela.draga@unipd.it; 2 Department of Earth and Environmental Sciences, University of Milano-Bicocca, 20126 Milano, Italy; sergio.sgorbati@unimib.it

**Keywords:** *Puya raimondii*, plant reproduction systems, plant population genetics, fixation index, Antonio Raimondi

## Abstract

*Puya raimondii* Harms is a charismatic species discovered in the Cordillera Blanca (now Huascarán National Park, Peru) in 1867 by the great Italian-born Peruvian geographer and naturalist Antonio Raimondi. The importance of this plant is due to its imposing size, the rare and extreme ecosystem that depends on it, and the fact that it is linked to the name Antonio Raimondi. Four studies on its genetic diversity revealed a range of patterns, with a fixation index of 0.740 as weighted mean and gene flow as low as 0.02–0.03. In fact, the vast majority of the total genetic variation was documented between populations, with very low genetic variation found within populations (weighted mean genetic diversity as low as *Hs* = 0.072 and mean genetic similarity very high, ranging from 96% up to 99%). We hypothesize that the narrow genetic base of *P. raimondii* populations may be due to a combination of factors: (i) an inbreeding-based reproductive strategy (i.e., mating between individuals related by common ancestry), which leads to homozygosity and genomic uniformity; (ii) strong environmental selective pressure (e.g., day–night temperature excursion, long dry period, etc.), which favors only the highest fitness individual genotypes; and (iii) a long life cycle, which hampers recombination events and reduces genetic diversity. Overall, these factors suggest that *P. raimondii* is a genetically fragile, fragmented, and endangered species.

## 1. Introduction

*Puya raimondii* Harms is a charismatic species that was discovered and described in the Cordillera Blanca (now Huascarán National Park, Peru) by Antonio Raimondi in 1867. The importance of this plant is due to its imposing size (it is the largest member of the Bromeliaceae family), the rich and uncommon ecosystem that depends on it, and the fact that it is linked to the name of Antonio Raimondi, who was born in Milan in 1824 and emigrated to Peru in 1850. In 1851, he became a professor of natural history in the Collegio de la Independencia, but most importantly, in 1856, he was one of the professors who founded the medical school at the National University of San Marcos, and in 1861, he founded the Department of Analytical Chemistry. This article is dedicated to his memory since this year marks the two-hundredth anniversary of his birth.

Notably, *P. raimondii* is a high-altitude species from the Andes, ranging from 3000 to 4800 m [1]. It is colloquially known as the Queen of the Andes and reproduces only once in a lifetime (i.e., semelparity), with plants usually flowering at 40–100 years of age [2,3]. Consequently, *P. raimondii* is considered genetically fragile, fragmented and endangered because of its long generation time and death after its first reproduction, which are biological features that prevent genetic diversity [1]. Thus, the conservation of *P. raimondii* populations is of paramount importance, as the impact of climate change and human activity, including the exploitation of plants for various purposes, can potentially lead to the deterioration of these unique populations, which possess a rich historical and distinctive evolutionary legacy.

The aim of this work is to critically review the main studies carried out on the population genetics of *P. raimondii* to infer its reproductive systems and hence to provide a solid basis for drafting an adequate plan for the conservation of this fascinating plant and its extraordinary ecosystem. We are confident that such a plan must start from the local populations representing the genetic material best preserved in protected areas, which have experienced less genetic erosion and have been shown to be highly divergent, highly inbred and characterized by high genetic loads [4]. This genetic material should constitute the starting point for the reinforcement of the species within its original distribution area, which would allow for the replacement of the small populations destined to disappear and ensure the conservation of its characteristic ecosystem. Such an action, carried out by the Peruvian universities along with national and regional institutions, would also constitute a dutiful homage, on the two hundredth anniversary of his birth, to the great Italian geographer and naturalist to whom *P. raimondii* has linked its name as well as the Peruvian history of the past two centuries.

## 2. Population Genetics of *P. raimondii*

To date, four studies have been conducted to assess the genetic diversity of *P. raimondii* over a period of two decades [4,5,6,7]. The initial study, conducted by Sgorbati et al. [5], employed amplified fragment length polymorphism (AFLP), random amplification of polymorphic DNA (RAPD) and chloroplast simple sequence repeats (cpSSR) markers to assess the genetic structure of 160 individuals sampled from eight distinct populations. Among the 217 AFLP markers used, 7 were polymorphic within populations, and 18 were polymorphic among populations, scoring a total genetic diversity (*Ht*) of 0.295 (Table 1). Notably, four populations were entirely monomorphic, whereas the remaining populations presented only one to three polymorphic markers. This was reflected in a within-population genetic variation of less than 4% and therefore high genetic differentiation among populations, accounting for 96.1% of the total variation, i.e., *Fst* = 0.961 (Table 1). The estimated gene flow equal to *Nm* = 0.02 (Table 1) was very low, suggesting nearly complete genetic isolation between populations, highlighting the restricted dispersal of genetic materials (pollen, seed, and plantlet sources) and strong geographic barriers.

In this study, to further explore the genetic background of *P. raimondii*, RAPD markers were used to analyze a progeny set of 13 plants. Only 2 out of 63 parental loci were heterozygous, suggesting a high homozygosity level of 96%. Additionally, no polymorphisms were detected with seven universal cpSSR primers. In terms of reproductive biology, the consistent ploidy across seeds and leaves excluded apomixis as the cause of high homozygosity, suggesting instead that the species predominantly reproduce via autogamy. These findings provide the first insights into the genetic diversity among *P. raimondii* populations through the assessment of molecular markers. Furthermore, information was gleaned regarding its reproductive biology via genome size estimation and ploidy level analysis [5].

**Figure 1:**
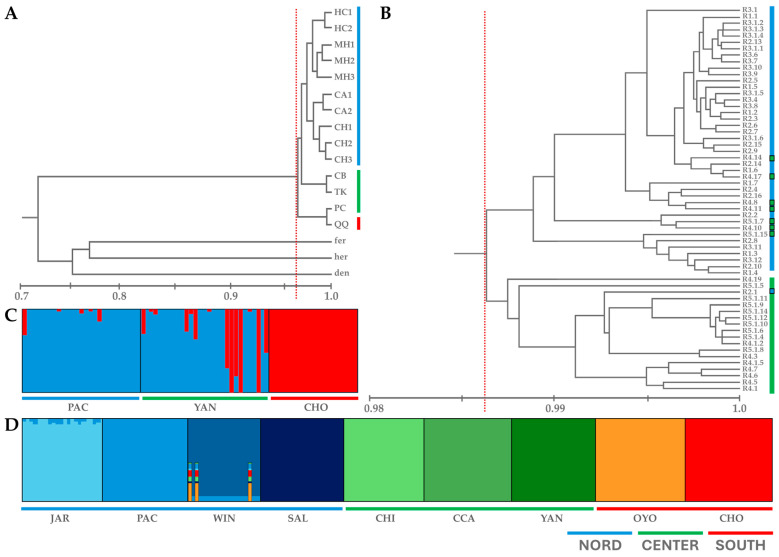
Graphical illustration of the reported genetic data from the four studies in question. (**A**) UPGMA dendrogram displaying the genetic similarity among populations of *P. raimondii* from the work of Sgorbati et al. [5]. Abbreviations of the populations are given in Table 2, while the three other species of *Puya* are included as outgroups: fer-*P. ferruginea*, her-*P. herrerae*, and den-*P. densiflora*. (**B**) UPGMA dendrogram of the genetic similarity estimates from Hornung-Leoni et al. [6], including the individuals from five populations reported as R1 to R5, followed by the genotype number. (**C**) STRUCTURE analysis, assuming the number of clusters (K) = 2 of the three populations (abbreviations as in Table 2), of *P. raimondii* from the study of Tumi et al. [7]. (**D**) STRUCTURE analysis of K = 9 (as the optimal solution) of the nine populations analyzed by Liu et al. [4] (for population abbreviations, see Table 2) and the well-defined regional divisions between the northern, central, and southern regions in the corresponding colors: blue, green and red.

A decade later, Hornung-Leoni et al. [6] investigated five populations of *P. raimondii* from two localities, Huascarán and Canchayllo (Table 2), using AFLP markers with four primer combinations for DNA fingerprinting. This study, which included a total of 60 individual plants, presented results that apparently contrast with the findings of Sgorbati et al. [5]. Here, 32.1% of the genetic variation occurred among populations, whereas 67.9% was found within populations. This distribution suggests that a significant proportion of the total genetic diversity, corresponding to *Ht* = 0.230 (Table 1), was retained within populations [6]. To summarize, this study suggests that high genetic variation is found within populations, even though the UPGMA dendrograms generated from AFLP data via the Dice index [9] shared similar patterns in terms of genetic similarity statistics with those of the study of Sgorbati et al. [5] (Figure 1A,B). In the study of Hornung-Leoni et al. [6], the estimates of genetic similarity ranged from approximately 98 to 99% (red line, Figure 1B), with an admixed cluster of individuals from the five populations included in the analysis. These values were even higher than those reported by Sgorbati et al. [5], who reported approximately 96% genetic similarity and included three other species of *Puya* as outgroups: *Puya ferruginea* (R. and Pav.) L. B. Sm., *Puya herrerae*, and *Puya densiflora* (Figure 1A).

In contrast, Tumi et al. [7,10] utilized SSR markers to examine the genetic structure of *P. raimondii*, analyzing 84 individuals from three populations (Figure 1C, Table 2). That study revealed a mean of 0.051 for the observed heterozygosity and a higher expected heterozygosity within populations, that is, *Hs* = 0.217 (Table 1), indicating a significant excess of homozygosity. Moreover, they reported 65% genetic variation among populations and only 35% within populations. The inbreeding coefficient (*Fis* = 0.776) and fixation index (*Fst* = 0.662) reported in Table 1 indicate substantial inbreeding, along with significant genetic differentiation among populations [7]. The overall results of this study are in accordance with the findings of Sgorbati et al. [5]. However, Tumi et al. [7] drew attention to certain inconsistencies between their data and those reported by Sgorbati et al. [5] and Hornung-Leoni et al. [6]. In particular, they proposed that the discrepancies observed among these studies could be attributed to the specificity of the genetic markers and to differences in the age of the sampled plants, emphasizing that their analyses were based on juvenile plants, while those performed by Sgorbati et al. [5]. and Hornung-Leoni et al. [6] were founded on adult plants, although none of the authors provided information on the size or age of the plants sampled. In this context, it is important to highlight that the age of the plants is irrelevant when employing genomic DNA-based markers, such as AFLP and SSR ones, given that these molecular markers are not affected by the age or developmental stage of the individual plants.

Most recently, Liu et al. [4] estimated the genetic diversity of 200 individuals sampled from nine populations of *P. raimondii* via whole-genome sequencing. Approximately 95.65% of the *P. raimondii* sequence reads were accurately mapped to the reference genome. The results revealed very low genetic diversity within populations and high genetic divergence among them. The *Fst* values ranged from 0.88 to 0.92 (Table 1), indicating strong genetic isolation. Population structure analyses clearly divided the nine populations, with all individuals being genetically assigned to their respective geographic groups (Figure 1D).

Overall, the fixation index calculated over all four studies was equal to *Fst* = 0.771 as the weighted mean, ranging from a minimum of 0.144 [6] to a maximum of 0.961 [5], and the most reliable of those statistics is based on the whole-genome sequencing recently reported by Liu et al. [4]. In fact, the proportion of genetic diversity among populations was as high as *Fst* = 0.88–0.92. Hence, most of the total genetic variation was documented among populations, with very low genetic variation found within populations (i.e., weighted mean as low as *Hs* = 0.072). The high genetic differentiation among populations was confirmed by gene flow estimates, which revealed values as low as *Nm* = 0.02–0.03 [4,5].

**Figure 2:**
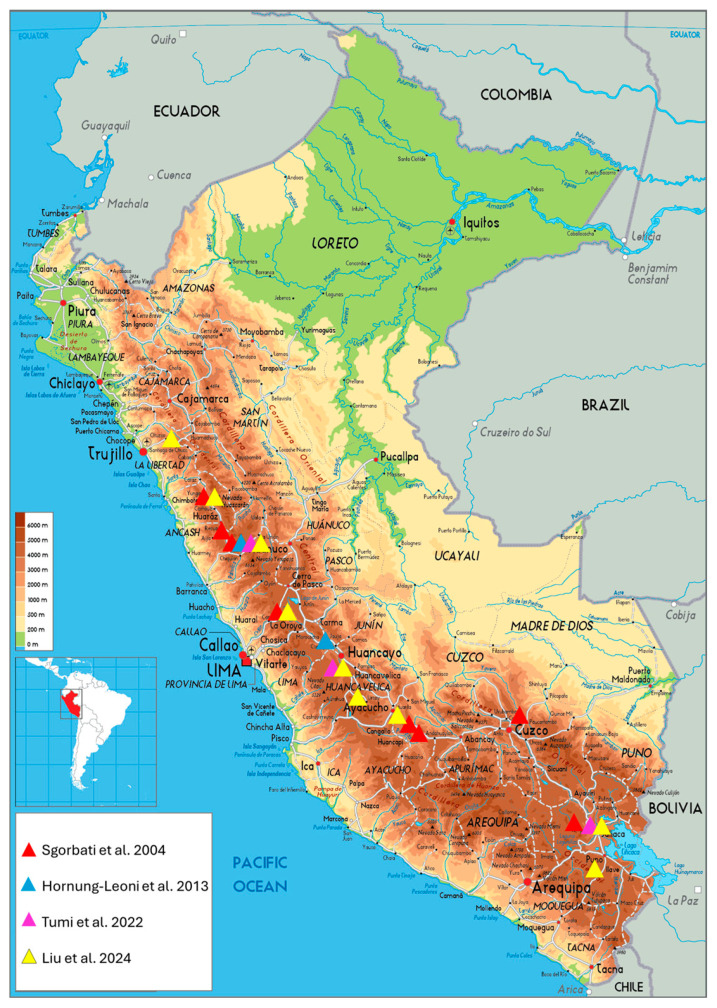
Geographical distribution of *P. raimondii* populations sampled in the Peruvian Andes, according to the four reported studies [4,5,6,7].

The different methodologies used across these studies, i.e., AFLP, SSR, and WGS, may account for some of the variations in the reported data. Molecular markers are widely integrated and employed in genetic diversity assessment for ease and efficiency of use [11,12,13,14,15]. In particular, the AFLP markers proved reliable in our case and yielded results similar to those obtained by new technologies such as WGS, e.g., the studies of Sgorbati et al. [5] and the work of Liu et al. [4] 20 years later. Moreover, the number of representative populations included in these studies (Table 2), on the basis of their geographical distribution across the northern, central, and southern regions of the Peruvian Andes (Figure 1 and Figure 2), may also influence the divergence in the reported results. For example, the study of Hornung-Leoni et al. [6] included five populations from two geographical locations, the northern and central regions of the Andes (blue triangles, Figure 2), in accordance with the reported UPGMA dendrogram (Figure 1B). Although the study analyzed five *P. raimondii* populations, the AMOVA results referred to data from “nine populations of both species of Andean *Puya*”, creating confusion regarding the actual number of populations and species included in the genetic diversity assessment data.

On the other hand, the remaining three studies sampled populations from the northern, central, and southern regions of the Peruvian Andes, with the study of Tumi et al. [7] representing three populations from each geographic zone (Figure 2). In this study, the central Yanacancha (YAN) population presented the same membership in the STRUCTURE analysis as the northern Pacahapaqui (PAC) population did (Figure 1C). These two populations, along with the other seven populations included in the study by Liu et al. [4], were clearly delineated by the STRUCTURE analysis (Figure 1D) and attributed to their respective geographic zones, i.e., north, center, and south, according to the reported lower K values (K = 3; see Liu et al. [4]). 

In addition to the geographical distribution and representativeness of *P. raimondii* populations, it is also crucial to consider the methodology adopted for sampling individuals within each site. In the study by Sgorbati et al. [5] leaf samples for genomic DNA isolation were collected from relatively well-spaced plants, i.e., at least 100 m apart, within each of the eight populations investigated, while in the study of Tumi et al. [7] the individuals of the three populations analyzed were sampled at a minimum distance of 10 m. It is not known which distance can be considered sufficient to collect different mother plants and distinct progeny plants in *P. raimondii*. We are aware that spatial sampling protocol is crucial to understanding population genetics and very useful to interpret inbreeding rates and genetic diversity patterns. Some information is available for *Puya hamata*, a different species phylogenetically and ecologically similar to *P. raimondii*. For instance, Rivadeneira et al. [16] demonstrated that poor capacity of seed dispersal and territoriality in hummingbird pollinators potentially create patterns of genetic diversity at relatively fine scales. Sometimes a mother plant produces very large offspring of daughter plants, a phenomenon which by itself reduces genetic diversity among plants within subpopulations. This is compounded further by territorial hummingbirds which restrict gene flow to the closest plants and hamper gene flow among the more distant ones (Paul Ramsay, pers. comm.). As a matter of fact, Rivadeneira et al. [16] found clear patterns relating to these influences in the genetic diversity of *P. hamata*.

In fact, the strong geographic isolation of *P. raimondii* populations across the Peruvian and Bolivian Andes, whose plants are found solely approximately 4000 m above sea level (“sky-islands” distribution pattern), seems to efficiently hamper any gene flow among populations by means of either animal-based pollen and seed dispersal or human-mediated plant migration and introduction.

To summarize, from a genetic perspective, Sgorbati et al. [5] and Tumi et al. [7] reported high genetic differentiation among populations, with minimal within-population diversity, reinforcing the idea that *P. raimondii* populations may experience limited gene flow and significant genetic isolation due to geographic distribution (Figure 2). In contrast, Hornung-Leoni et al. [6] reported higher within-population genetic diversity and moderate genetic differentiation, implying greater connectivity among populations. The most recent study by Liu et al. [4] provided a more comprehensive assessment of the genetic diversity in this species through high-resolution genomic data, resolving some of the discrepancies reported by earlier studies. These findings confirmed low genetic diversity within populations and strong genetic isolation among populations, as indicated by high *Fst* values (Table 1), and confirmed the genetic diversity results of Sgorbati et al. [5]. The overall pattern indicates that *P. raimondii* is a genetically fragile, fragmented and endangered plant species because of its long generation time and death after its first reproduction, which are biological features that prevent genetic diversity within populations and increase genetic isolation and differentiation among populations.

Interestingly, to shed light on the demographic history of this unique species, Liu et al. [4] conducted a detailed investigation by analyzing the genomes of *P. raimondii* and its close relative, an iteroparous species, *Puya macrura*. It is hypothesized that the two species diverged approximately 4.7 million years ago and experienced a bottleneck 1–0.7 million years ago during the Pleistocene. Here, the population size of both species decreased, likely due to climatic events, even if they evolved differently, with *P. raimondii* being more sensitive to climate challenges than *P. macrura* was due to its low adaptability rate and semelparity reproductive strategy. In contrast, *P. macrura* adapted rapidly and managed to recover and expand [4]. These findings are highly relevant given that they reflect the cost of the low genetic diversity present in *P. raimondii*.

The case study of *P. raimondii* presented here deserves a further discussion focused on the inbreeding-based reproductive strategy of the species (i.e., mating between individuals related by common ancestry). Molecular marker data suggest that we are dealing with individuals and populations that at the species level are most likely characterized by a high degree of homozygosity stemmed from inbreeding (i.e., mediated by either selfing or outcrossing and sib-mating or promoted by mixed conditions). It is well known that inbreeding usually has negative effects on the evolution and preservation of a small lineage and gene pool [17,18].

In a population with a high degree of inbreeding, progenies are more likely to inherit harmful mutations from both germ lines. Therefore, plants carrying these mutations with an inbreeding-based reproductive strategy can more quickly manifest lower vigor and poorer viability and/or fertility. Hence, progenies expressing these mutations may worsen their fitness and consequently become less likely for deleterious mutations to be passed on to subsequent generations. Such behavior seems particularly true in *P. raimondii* and boosted in this species as it typically has a very long life cycle, is exposed to a strong environmental selective pressure, and reproduces once every several dozens of years.

Recently, Fortier [19] published about the importance of *P. raimondii*, focusing on a call for more actions for its conservation through in situ and ex situ initiatives. It is, therefore, possible that future climatic challenges could prove fatal for this species, since it may reduce the populations of *P. raimondii* by one-fifth, or even by half, as reported by Fortier [19], or alternatively, by 45% [20]. *P. raimondii* plants are subject to a number of anthropogenic risks, including the use of fire for land regeneration and the avoidance of domesticated animal entrapment in its sharp leaves [19,21]. Furthermore, its gigantic and resilient leaves are utilized as a food source attained by foraging [21]. In addition to anthropogenic risks, Suni et al. [22] documented the impact on *P. raimondii* and the surrounding ecosystem of fires occurring every 4–6 years, coinciding with periods of drought and low temperatures preceded by high precipitation [22]. These recent studies in 2024 are of paramount importance, as they correlate genetic information [4], environmental issues [22], and conservation actions [19] to more accurately identify the importance and necessity for the risk assessment of *P. raimondii*.

Naturalists and scientists hold great responsibility for discovering and transmitting data on rare and endangered species, leading to pivotal actions for the protection of not only the species but also the surrounding ecosystem. The high Andes region is home to some of the most biodiverse ecosystems on the planet, and as such, it is highly conserved [19,23,24]. However, the importance of acquiring genetic data and evaluating emerging technologies is as crucial as the accurate interpretation and transmission of these data. To this end, the standardization of genetic data for the *P. raimondii* population structure is of paramount importance for the implementation of protective measures for this giant yet fragile species, known as the Queen of the Andes.

## 3. Molecular Techniques for Genetic Diversity Analysis of *P. raimondii*

Molecular markers and, more generally, genomics data are very useful for studying plant reproductive biology as well as mating systems (for example, degree of selfing vs. outcrossing or sexuality vs. apomixis) and their direct consequences for population genetics (i.e., heterozygosity vs. homozygosity, population structure, genetic diversity partitioning, inbreeding levels, genetic drift dynamics, etc.) and for assessing genetic diversity within and genetic differentiation among populations to infer basic information on plant reproductive patterns. In the last 10 years, our research group has applied molecular markers to several crop and model species for genotyping individuals and populations by amplifying target regions or by sequencing whole genomes, including cereals (corn, barley, wheat, etc.) [25,26,27], vegetables (lettuce, chicory, endive, sweet potato, fennel, common bean, etc.) [28,29,30,31,32,33], fruit trees (olive, grapevine, pear, etc.) [34,35,36] and medicinal herbs (*Hypericum* and *Cannabis*) [37,38]. Genetic diversity/similarity statistics as well as inbreeding coefficients and differentiation statistics are useful not only for characterizing populations and preserving germplasm resources but also for breeding or protecting plant varieties and food derivatives.

**Figure 3:**
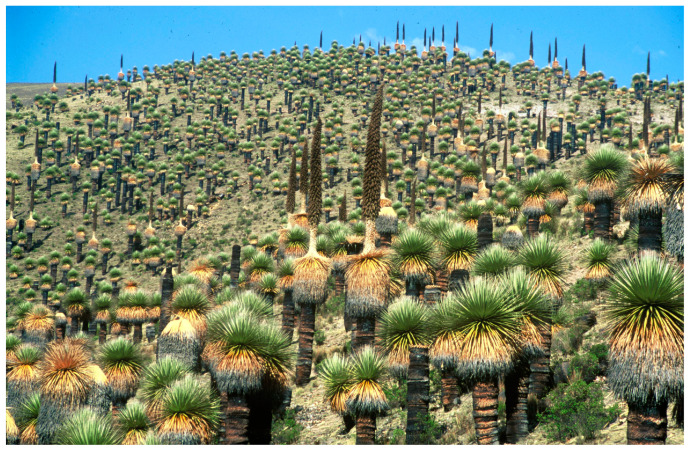
Fascinating *P. raimondii* individuals of the Titancayocc population in Ayacucho, Peru.

In addition to conventional, well-established and largely studied crop plants, we also focused our attention on neglected, unknown or uncommon (nonfood and nonfeed) species, including *P. raimondii* Harms. It is a fascinating plant species whose populations live in a rare and unique ecosystem in the Andes, between 3.200 and 4.400 m of altitude, in the Puna environment, where only rare, relict *Polylepis* ssp. and *BuddlIeja* ssp. woods have escaped felling over the course of a thousand years. Individual plants are exceptionally large, and the species is known as the largest of the Bromeliaceae family (Figure 3). Genetic information on this species is scarce, and its reproductive biology is completely unexplored.

The genetic variability or uniformity of *P. raimondii* populations so far characterized using molecular markers [4,5,6,7] were assessed by the calculation of genetic similarity coefficients [39,40]. The standard genetic diversity (*H*) and genetic differentiation (*D*) statistics of Nei [41] and the inbreeding (*F*) coefficients of Wright [42] were also used to summarize the genetic structure of *P. raimondii* populations and the distribution of genetic variation using molecular markers. These overall statistics available for *P. raimondii* are reported in Table 1.

All sets of genetic diversities or similarities calculated for *P. raimondii* populations were analyzed to assess within- and between-population genetic variation, particularly in terms of individual plant contributions to the total genetic variability. Each of the symmetrical matrix of genetic distances or similarities calculated from marker allele frequencies and multilocus fingerprints or genotypes was used to construct UPGMA dendrograms and develop PCA centroids of *P. raimondii* populations [5,6,7]. The STRUCTURE analysis was also used for ancestry group reconstruction in *P. raimondii* [4,7]. These graphical representations referred to *P. raimondii* are reported in Figure 1.

Sgorbati et al. [5] conducted pioneering research on the genetic diversity and reproductive biology of *P. raimondii* populations in the Peruvian Andes (Figure 2). The data provided by this work led the International Union for the Conservation of Nature (IUCN) to formulate an assessment of the risk of extinction of this species [1]. In contrast, it is not listed in the most recent Red List for Andean plants of Bolivia [19,43]. Despite this, all genetic-oriented studies have been conducted exclusively in Peru.

Subsequently, further studies on the plant genomics and reproductive genetics of this species have been conducted by a few research groups [4,6,7].

Basic information on the *P. raimondii* populations sampled and analyzed thus far is reported in Table 2. For each of the populations investigated by Sgorbati et al. [5], Hornung-Leoni et al. [6], Tumi et al. [7], and Liu et al. [4], the information included the number of plants, the name of the location and the geographic location.

Overall, data from molecular marker-based fingerprinting and whole-genome sequencing were used to understand the population genetic structure of *P. raimondii* and infer its reproductive system biology. In fact, the vast majority of the total genetic variation was documented between populations, with very low genetic variation found within populations (mean estimates of genetic similarity across all populations varied from approximately 96% to 99% or 100%, i.e., genetic identity).

## 4. Conclusions

Our paper critically reviews the population genetics of the Queen of the Andes (*P. raimondii*), an iconic species here considered as a case study. The comprehensive analysis of the available molecular markers and genomics data enabled a reappraisal of the genetic diversity patterns in *P. raimondii* and provided a solid basis for drafting and applying an adequate plan for the conservation of *P. raimondii*, as this outstanding giant rosette bromelia plant was already considered the endangered Queen of the Andes by Sgorbati et al. [5] two decades ago.

Here, we hypothesize that the narrow genetic base of *P. raimondii* populations may be due to a combination of biological and environmental factors: (i) an inbreeding reproductive strategy, which leads to homozygosity and genomic uniformity; (ii) strong environmental selective pressure (e.g., day–night temperature excursion up to 30 °C, long dry period, etc.), which favors only the highest-fitness individual genotypes; and (iii) a long life cycle, which hampers recombination events and consequently reduces genetic diversity.

The key findings derived from the available genomics data are consistent with evidence on the population genetics of *P. raimondii*: most of the populations of *P. raimondii* studied so far proved to be highly divergent, genetically inbred, and characterized by random genetic drift, absent flow of genetic material and a high genetic load. This translates into low genetic diversity of populations, which result in genetic isolation, contributing to the fragmentation of the species. Overall, these findings are critical for conservation efforts. There are populations, mostly growing in protected areas and consisting of many thousands of individuals, which appear to retain considerable fitness, given that they reproduce regularly and have a high proportion of juvenile individuals. These populations could provide genetic material, in the form of seedlings grown for a few years in nurseries, to strengthen existing populations or form new ones within the distribution area of the species, where many small populations are at risk of disappearing.

In conclusion, on the basis of the available population genetics data, *P. raimondii* is confirmed to be a genetically fragile, fragmented and endangered plant species, which deserves urgent conservation actions.

## Figures and Tables

**Table 1:** Genetic diversity statistics of *Puya raimondii* reported by the four studies. The number of populations and genotypes and the type of DNA analysis used for the assessment of molecular polymorphisms at the genome level. Statistical data include total genetic diversity (*Ht*), expected mean heterozygosity within each population (*Hs*), genetic differentiation between populations (*Dst*), the fixation index (*Fst*), the inbreeding coefficient (*Fis*) and gene flow (*Nm*) estimates.

	No.	No.	DNA	Genetic Diversity Statistics *					
Authors	Pops	Genotypes	Analysis	*Ht*	*Hs*	*Dst*	*Fis*	*Fst*	*Nm*
Sgorbati et al. [5]	8	160	AFLP	0.295	0.011	0.284	n.a.	0.961	0.02
Hornung-Leoni et al. [6]	5	60	AFLP	0.230	0.197	0.033	n.a.	0.144	2.97
Tumi et al. [7]	3	84	SSR	0.378	0.217	0.161	0.776	0.662	0.13
Liu et al. [4]	9	200	WGS	0.114	0.009	0.105	0.700	0.88–0.92	0.02–0.03

* Sgorbati et al. [5] and Hornung-Leoni et al. [6] calculated genetic diversity statistics of dominant AFLP molecular marker data. Tumi et al. [7] applied codominant SSR markers or microsatellite markers, whereas Liu et al. [4] used SNP marker data derived from whole-genome sequencing (WGS). Notably, for Tumi et al. [7], *Ht*, *Dst* and *Nm* values were added via calculations on the basis of the *Hs* and *Fst* values reported, and for Liu et al. [4], the heterozygosity estimates for the subpopulation *Hs* were derived from the *Fst* index via nucleotide diversity statistics across all of the populations (i.e., species level), which were considered equal to *Ht*. In addition, the Fst (~Gst) value of 0.426 reported by Tumi et al. [7] was corrected by genetic diversity within populations (Hs) due to the high number of alleles per locus [8] as G’st = [Gst(1 + Hs)]/(1 − Hs) = [0.426(1 + 0.217)]/(1 − 0.217) = 0.662.

**Table 2:** Population sampling information on the geographic location of *P. raimondii*.

Authors	Population/Individuals	Location	Geographic Coordinates
Sgorbati et al. [5]	Huinchos (HC)/20	Pueblo Libre, Ancash	−9.1333°, −77.8667°
	Minas Huinac (MH)/20	Aija, Ancash	−9.7000°, −77.6667°
	Carpa (CA)/20	Recuay, Ancash	−9.8833°, −77.2833°
	Cerro Huaypian (CH)/20	Huaros, Lima	−11.3833°, −76.5333°
	Pampa-corral (PC)/20	Lares, Cusco	−13.1500°, −71.9833°
	Carabamba (CB)/20	Chiara, Ayacucho	−13.4500°, −74.1333°
	Titankayocc, (TK)/20	Vischongo, Ayacucho	−13.5667°, −73.9833°
	Quello Quello (QQ)/20	Lampa, Puno	−15.2500°, −70.3500°
Hornung-Leoni et al. [6]	R1/7	N.P. Huascarán	−9.87697°, −77.27311°
	R2/14	N.P. Huascarán	−9.88333°, −77.25722°
	R3/14	N.P. Huascarán	−9.89056°, −77.28000°
	R4/13	Canchayllo	−11.83225°, −75.71535°
	R5/10	Canchayllo	−11.83000°, −75.69556°
Tumi et al. [7]	Yanacancha (YAN)/15	Cachi, Chupaca, Yanacancha	−12.247°, −75.475°
	Yanacancha (YAN)/14	Huáscar, Chupaca, Yanacancha	−12.236°, −75.440°
	Pacahapaqui (PAC)/28	Bolognesi, Aquia	−9.958°, −77.088°
	Lampa (CHO)/27	Choconchaca, Lampa	−15.258°, −70.088°
Liu et al. [4]	OYO/23	Ichuna, General Sanchez Cerro, Moquegua	−16.167°, −70.5825°
	CHO/23	Lampa, Lampa, Puno	−15.2581°, −70.0883°
	CHI/22	Chiara, Huamanga, Ayacucho	−13.2743°, −74.2054°
	CCA/23	Huancavelica, Huancavelica	−12.8254°, −75.0678°
	YAN/23	Cachi and Huáscar, Junin, Chupaca, Yanacancha	−12.2471°, −75.4755°
	JAR/22	Huaros, Canta, Lima	−11.3913°, −76.5601°
	PAC/23	Bolognesi, Aquia, Ancash	−9.958°, −77.0881°
	WIN/22	Huaylas, Pueblo Libre, Ancash	−9.1059°, −77.8677°
	SAL/22	Otuzco, Salpo, La Libertad	−8.0685°, −78.5744°

## Data Availability

All genetic diversity statistics here presented and discussed are derived and reviewed from the original data reported on the cited *Puya raimondii* manuscripts.
